# The Efficacy and Safety of Bivalirudin Versus Heparin in the Anticoagulation Therapy of Extracorporeal Membrane Oxygenation: A Systematic Review and Meta-Analysis

**DOI:** 10.3389/fphar.2022.771563

**Published:** 2022-04-14

**Authors:** Min Ma, Shichu Liang, Jingbo Zhu, Manyu Dai, Zhuoran Jia, He Huang, Yong He

**Affiliations:** ^1^ Department of Cardiology, West China Hospital of Sichuan University, Chengdu, China; ^2^ Department of Cardiology, The Sixth People’s Hospital of Chengdu, Chengdu, China; ^3^ Department of Cardiology, Union Hospital, Tongji Medical College, Huazhong University of Science and Technology, Wuhan, China; ^4^ Department of Cardiology, The First Affiliated Hospital of Anhui Medical University, Hefei, China

**Keywords:** extracorporeal membrane oxygenation, heparin, bivalirudin, meta-analysis, systematic review

## Abstract

**Background:** Bivalirudin is a direct thrombin inhibitor (DTI) that can be an alternative to unfractionated heparin (UFH). The efficacy and safety of bivalirudin in anticoagulation therapy in extracorporeal membrane oxygenation (ECMO) remain unknown.

**Methods:** This study followed the preferred reporting items for systematic reviews and meta-analyses (PRISMA) guidelines. A systematic literature search was performed in PubMed, EMBASE, and The Cochrane Library databases to identify all relevant original studies estimating bivalirudin’s efficacy and safety versus UFH as anticoagulation therapy in ECMO. The time limit for searching is from the search beginning to June 2021. Two researchers independently screened the literature, extracted data and evaluated the risk of bias of the included studies. The meta-analysis (CRD42020214713) was performed *via* the RevMan version 5.3.5 Software and STATA version 15.1 Software.

**Results:** Ten articles with 847 patients were included for the quantitative analysis. Bivalirudin can significantly reduce the incidence of major bleeding in children (*I* ^*2*^ = 48%, *p* = 0.01, odd ratio (OR) = 0.17, 95% confidence interval (CI): 0.04–0.66), patient thrombosis (*I* ^*2*^ = 0%, *p* = 0.02, OR = 0.58, 95% CI: 0.37–0.93), in-circuit thrombosis/interventions (*I* ^*2*^ = 0%, *p* = 0.0005, OR = 0.40, 95% CI: 0.24–0.68), and in-hospital mortality (*I* ^*2*^ = 0%, *p* = 0.007, OR = 0.64, 95% CI: 0.46–0.88). Also, comparable clinical outcomes were observed in the incidence of major bleeding in adults (*I* ^*2*^ = 48%, *p* = 0.65, OR = 0.87, 95% CI: 0.46–1.62), 30-day mortality (*I* ^*2*^ = 0%, *p* = 0.61, OR = 0.83, 95% CI: 0.41–1.68), and ECMO duration in adults (*I* ^*2*^ = 41%, *p* = 0.75, mean difference (MD) = −3.19, 95% CI: −23.01–16.63) and children (*I* ^*2*^ = 76%, *p* = 0.65, MD = 40.33, 95% CI:−135.45–216.12).

**Conclusions:** Compared with UFH, bivalirudin can be a safe and feasible alternative anticoagulant option to UFH as anticoagulation therapy in ECMO, especially for heparin resistance (HR) and heparin-induced thrombocytopenia (HIT) cases.

## Introduction

Extracorporeal membrane oxygenation (ECMO) is a life-supporting system that provides circulatory and/or pulmonary support for patients suffering from severe, life-threatening disease (Karagiannidis et al., 2016), including refractory acute heart failure, ST-segment elevation myocardial infarction (STEMI), or acute respiratory distress syndrome (ARDS). Moreover, ECMO is applied in severe conditions, such as heart transplantation and shock, as well. With the development of medical technology, ECMO complications have reduced significantly, with greatly improved survival rates. In recent studies, ECMO proved its superiority in reducing the mortality in patients with severe respiratory failure from COVID-19 (Shaefi et al., 2021). However, during ECMO treatment, coagulation-related complications (i.e., bleeding or thrombosis) remain the main factors affecting morbidity and mortality. Therefore, clinical researches has focused on the avoidance of those complications.

Blood’s exposure to a foreign surface may render patients vulnerable to thromboembolic events, which can be prevented by the heparinization of blood (Finley and Greenberg, 2013). For decades, unfractionated heparin (UFH) has been the most common anticoagulant and mainstay antithrombotic in ECMO. Nevertheless, its clinical use may be restricted by UFH-related complications, such as heparin resistance (HR), caused by the consumptive deficiency of antithrombin (AT III), and heparin-induced thrombocytopenia (HIT). This devastating event may occur with heparin exposure (Ortel, 2009; Koster et al., 2013). Therefore, replacement of anticoagulation therapy appears crucial.

Bivalirudin is an alternative anticoagulant option. As an oligopeptide analog of hirudin, bivalirudin is a parenteral direct thrombin inhibitor (DTI), inherently independent of AT III. Moreover, bivalirudin is a bivalent DTI that binds specifically to thrombin at two sites without a cofactor (Warkentin et al., 2008). Furthermore, the reversible and transient binding to thrombin makes it a mainstream anticoagulant in the cardiac catheterization room (Warkentin et al., 2008). However, there are no large-scale, randomized controlled trials (RCTs) reporting the incidences of major bleeding, thrombosis, and mortality of bivalirudin versus UFH in the treatment of ECMO. Therefore, we believe it is worthwhile to carefully conduct a meta-analysis to evaluate the efficacy and safety of bivalirudin versus UFH in ECMO anticoagulation therapy.

## Methods

### Study Design and Literature Search

This is a registered meta-analysis on PROSPERO (https://www.crd.york.ac.uk/prospero/). The registration number is CRD42020214713.

The participants, intervention, comparison, outcome, and study design approach (PCIOS) were used to select clinical studies (Table 1). Reviews, meta-analyses, non-human studies, case reports, and conferences were excluded. Studies that did not compare the clinical outcomes between UFH and bivalirudin were excluded as well. Two authors (S. Liang and J. Zhu) independently searched the PubMed, EMBASE, and The Cochrane Library databases for articles published from inception until 1 June 2021, using the heading terms “heparin,” “unfractionated heparin,” “bivalirudin,” “extracorporeal membrane oxygenation,” “ECMO,” “ECMO treatment,” “ECLS,” or “ECLS treatment”. No language restrictions were used. The references of relevant literature were also searched to look for more eligible studies.

**TABLE 1:** ‘‘PICOS’’ approach for selecting clinical studies in the systematic search.

PICOS	
1 Participants	The patients (both adult and pediatrics) receiving the treatment of ECMO despite differences in ECMO indication and configuration, concurrent medications, and presence of HIT and HR.
2 Intervention	The patients who took bivalirudin during the treatment of ECMO.
3 Comparison	The patients who took UFH during the treatment of ECMO.
4 Outcomes	The incidence rate of major bleeding, thrombosis, and mortality
5 Study design	Prospective and retrospective observational studies; RCTs

ECMO, extracorporeal membrane oxygenation; HR, heparin resistance; HIT, heparin-induced thrombocytopenia; UFH, unfractionated heparin; RCT,randomized controlled trials.

### Data Extraction and Quality Assessment

Data were extracted by the same two independent readers (S. Liang and J. Zhu) who performed the literature search and study selection; the researchers were not blinded to the authors and institutions of included studies. Disagreements were solved by a third reader (M. Ma). Y. He supervised the whole process. This meta-analysis followed the guidelines for preferred reporting items for systematic reviews and meta-analyses (PRISMA) (Shamseer et al., 2015). The two reviewers extracted the following information independently: the first author, published year, study design (prospective/retrospective), study duration, total patients and number of patients in the bivalirudin and UFH groups, the doses in the bivalirudin and UFH group, and the incidence of thromboses, major bleeding, and mortality (per-patient).

For the observational studies, the Newcastle-Ottawa Scale (NOS) was used to assess the risk of bias. The NOS ranges from 0 (lowest) to 9 (highest), and studies with scores ≥6 are considered high quality. For RCTs, the modified Jadad quality scoring scale is used for the quality assessment, which includes the generation of random sequences, distribution methods, randomized concealment, double-blinding, withdrawals and dropouts. The Jadad score among four to seven is considered as good quality.

Sensitivity analysis of the included studies was conducted via a one-by-one elimination method to evaluate the meta-analysis’s stability. A Galbraith plot was used to find the cause of heterogeneity. Egger’s test was used to test the publication bias via Stata version 15.1 Software (The StataCorp LP, Texas City, United States).

### Statistical and Meta-Analysis

For studies describing the results via median and interquartile range (IQR), Standard deviations (SDs) of the mean differences (MDs) were obtained as described by former researches (Wan et al., 2014; Luo et al., 2018). The RevMan version 5.3.5 Software (The Cochrane Collaboration, Copenhagen, Denmark) and Stata version 15.1 Software (The StataCorp LP, Texas City, United States) was used for all statistical analyses. Statistical heterogeneity was assessed by using the Cochrane Q and the I square statistics. Heterogeneity was interpreted as absent (*I* ^*2*^:0–25%), low (*I* ^*2*^: 25.1–50%), moderate (*I* ^*2*^: 50.1–75%), or high (*I* ^*2*^: 75.1–100%) (Higgins et al., 2003). The use of a random-effects model was also considered when the number of studies was relatively small, and a random-effects model was applied to estimate the continuous outcome data for data with a *p*-value ≤0.1 and an *I* ^*2*^-value >50%, which indicated statistical heterogeneity (Higgins et al., 2003). Otherwise, a fixed-effects model was used. The overall log with its 95% CI was used as the summary of the overall effect size. A *p*-value <0.05 was considered statistically significant.

## Results

### Literature Search and Study Selection

**FIGURE 1:**
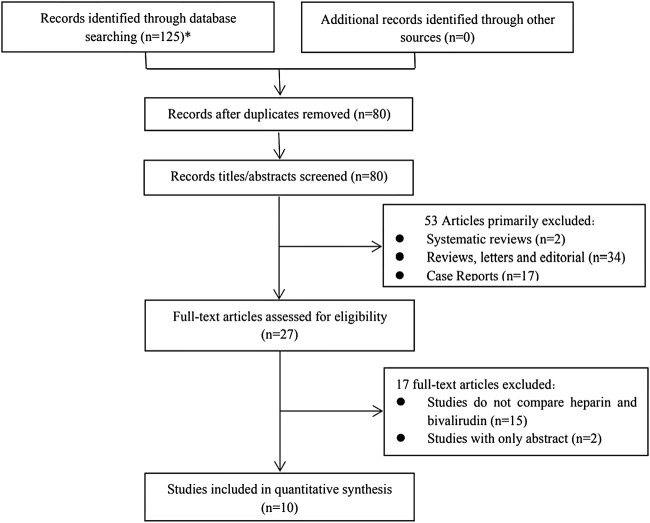
Flow chat of study selection (*101 from Embase, 20 from Pubmed and 4 from The Cochrane Library).

The literature search produced 125 total findings (101 on EMBASE, 4 on The Cochrane Library, and 20 on PubMed); 80 full texts were retrieved after duplicates were removed. The titles and abstracts of studies were screened, after which 53 articles were excluded due to the following reasons: systematic reviews (n = 2), reviews, letters and editorials (n = 34), case reports (n = 17). A total of 27 full-text articles were reviewed, and 17 were excluded later because they lacked the comparison between UFH and bivalirudin (n = 15) or they are only with abstracts (n = 2). Finally, ten unique retrospective observational studies (Ranucci et al., 2011; Pieri et al., 2013; Ljajikj et al., 2017; Berei et al., 2018; Macielak et al., 2019; Brown et al., 2020; Hamzah et al., 2020; Kaseer et al., 2020; Schill et al., 2021; Seelhammer et al., 2021) with 847 patients were included for the quantitative analysis. All articles were published before 1 June 2021. The literature screening process is presented in Figure 1.

### Data Extraction and Quality Assessment

Table 2 shows basic information from the included studies; Table 3 shows group definition of the bivalirudin group and clinical outcomes. Generally, these included studies met most NOS quality indicators. However, the control group of all the studies did not meet the standard of “community controls” and “no history of diseases” as the controls was from a hospital. Moreover, as the included studies were all case-control retrospective studies, they were not blinded to the case/control status. According to the NOS, all the included studies were considered as high quality (Supplemental Table S1).

**TABLE 2:** Basic information of the included studies.

Study	Duartion	Total Patients (Pediatric Patients)	VV/VA-ECMO	Indication of ECMO (Number of patients)	Heparin bolus	Bivalirudin group		Heparin group	
						Dose	Number (Pediatric Patients)	Dose	Number (Pediatric Patients)
Ranucci2011	January 2008-April 2011	21 (9)	NR	Postcardiotomy ECMO procedure (21)	100U/kg	0.03–0.05 mg/kg/h	13 (4)	5–10 U/kg/h	8 (5)
Pieri2012	January 2008-March 2011	20 (0)	10/10	NR	NR	0.025 mg/kg/h	10 (0)	3 U/kg/h	10 (0)
Ljajikj2017	March 2012-March 2016	57 (0)	NR	Left ventricular assist device implantation(57)	10 000 U	APTT>160s: 0.25 mg/kg/h	21 (0)	NR	36 (0)
						APTT<160s: 0.5 mg/kg/h			
Berei2018	January 2012 -September 2015	72 (0)	6/66	Cardiogenic shock (51)	80U/kg	0.04 mg/kg/h	44 (0)	8–12 U/kg/h	28 (0)
				Septic shock (11)					
				Respiratory shock (4)					
				Mixed shock (6)					
Macielak2019	January 2012 -June 2017	110 (0)	NR	Emergency salvafe (61)	50–100U/kg	0.01–0.1 mg/kg/h	10 (0)	12 U/kg/h	100 (0)
				Cardiogenic shock (46)					
				ARDS (29)					
				Respiratory insufficiency (29)					
				Failed to wean from CRB(23)					
				Others (12)					
Brown2020	March 2014-January 2018	15 (0)	7/5(3 peripheral RVAD)	NR	80U/kg	NR	NR	NR	NR
Hamzah2020	October 2014-May 2018	32 (32)	3/29	Heart transplantation (32)	50–100U/kg	Ccr>60 ml/min: 0.3 mg/kg/h renal impairment: 0.15 mg/kg/h	16 (16)	open chest:10U/kg/h <12M:18U/kg/h 1Y-12Y:16U/kg/h >12Y:14U/kg/h	16 (16)
Kaseer2020	January 2013-September 2018	52 (0)	24/28	Cardiogenic shock (13)	NR	0.1 mg/kg/h	19 (0)	10.4 U/kg/h	33 (0)
				Respiratory failure (13)					
				Heart and/or lung transplant (9)					
				Others (1)					
Schill2021	June 2018-December 2019	48 (0)*	16/32*	Postcardiotomy shock (16)*	50–100U/kg	Typical: 0.15 mg/kg/h	14 (0)*	20 U/kg/h or 28 U/kg/h	34 (0)*
				Respiratory failure (17)*		Ccr<30 ml/min: 0.075 mg/kg/h			
				Cardiogenic shock (15)*		Receiving CRRT: 0.1 mg/kg/h			
Seelhammer2021	January 2014-October 2019	422 (89)	64/358	Post cardiotomy (162)	1000U/kg	NR	134 (24)	NR	288 (65)
				Cardiac (100)					
				Respiratory (86)					
				Extracorporeal Cardiopulmonary					
				Resuscitation (69)					
				Post transplant (5)					

ARDS, acute respiratory distress syndrome; CPB, cardiopulmonary bypass; CRRT, continuous renal replacement therapy; VV-ECMO, Venovenous ECMO; VA-ECMO, Venoarterial ECMO; NR, Not reported; RVAD, right ventricular assist device. *runs.

**TABLE 3:** Definition of the bivalirudin group and clinical outcomes.

Study	Definition		
	Bivalirudin group	Thrombosis	Major Bleeding
Ranucci2011	Non-HIT patients	NR	NR
Pieri2012	Non-HIT patients	Thrombosis could be attributed either to the patient (ie, venous or arterial occlusion with clinical signs and symptoms or evident at the radiologic examination) or the oxgenator	NR
Ljajikj2017	HIT patients	NR	NR
Berei2018	Non-HIT patients	Clinically documented venous or arterial thromboembolism or thrombus within the ECMO circuit	Any bleeding event associated with a drop in hemoglobin of at least 3 mg/dl within the prior 24 h
Macielak2019	Non-HIT patients	Requirement for oxygenator exchange, requirement for circuit exchange, laboratory values indicating acute hemolysis (pfHg>50 mg/dl or LDH>1,000U/L), or systemic thromboembolism including VTE, intra-cardiac thrombus, or ischemic stroke	Clinically overt bleeding associated with a hemoglobin fall of at least 2 g/dl in a 24-h period or a transfusion requirement of one or more 10 ml/kg PRBC transfusions over that same time period
Brown2020	Non-HIT patients	Ischemic cerebral vascular accidents, ischemic digits, visceral ischemia, or pump failure due to suspected thrombosis	Intracranial hemorrhage, decrease in hemoglobin by 3 g/dl over 24 h in the setting of a bleed with overt source, hemodynamic instability with associated blood transfusion, fatal bleeding, and bleeding requiring an intervention such as epistaxis requiring nasal packing, GI bleeding with cauterization or clipping, washoutetc.
Hamzah2020	Non-HIT patients	Significant thrombosis is defined as thromboembolic events to the brain, visceral organs, or extremities. Circuit thrombosis that leads to circuit change is considered significant thrombosis	Bleeding associated with a decrease in the measurement of hemoglobin by 2 g/d or transfusion of packed RBCs at a rate greater than 20 ml/kg over 24 h. CNS bleeding or bleeding that requires surgical intervention would also be considered a significant bleeding event
Kaseer2020	Non-HIT patients and HIT patients	Composite thrombotic events postdecannulation defined as arterial and/or venous thromboembolism within 72 h of ECMO decannulation	Any bleed with a drop in hemoglobin of ≥3 mg/dl within 24 h
Schill2021	Non-HIT patients	Patients with a history of thrombophilia, arterial or venous thromboses, or circuit thromboses were considered to have high thrombotic risk	Patients with intracranial hemorrhage, bleeding requiring surgical intervention or massive transfusion were considered high bleeding risk
Seelhammer2021	Non-HIT patients	Ischemic complication (stroke, deep vein thrombosis, pulmonary, myocardial infarction, mesenteric ischemia)	NR

CNS, central nervous system; ECMO, extracorporeal membrane oxygenation; GI, gastro intestinal; HIT, heparin-induced thrombocytopenia; LDH, lactate dehydrogenase; NR, Not reported; PRBC, packed red blood cell; pfHg, plasma free hemoglobin; VTE, venous thromboembolism.

### Major Bleeding

Nine studies (Ranucci et al., 2011; Pieri et al., 2013; Ljajikj et al., 2017; Berei et al., 2018; Macielak et al., 2019; Brown et al., 2020; Hamzah et al., 2020; Kaseer et al., 2020; Schill et al., 2021) reported the incidence of major bleeding. Two studies (Macielak et al., 2019; Brown et al., 2020) were not included due to the different ways of expression (per ECMO day). The incidence rate of major bleeding is 0.223 and 0.139 per ECMO day in the UFH and bivalirudin group in Macielak et al. (Macielak et al., 2019)’s study, 0.308 and 0.062 per ECMO day in Brown et al. (Brown et al., 2020)’s study, respectively. Regarding Ljajikj et al. (Ljajikj et al., 2017)’s study, we considered both delayed chest closure and intracranial bleeding as major bleeding because the authors thought that delayed chest closure might also be the result of diffuse persisting bleeding.

**FIGURE 2:**
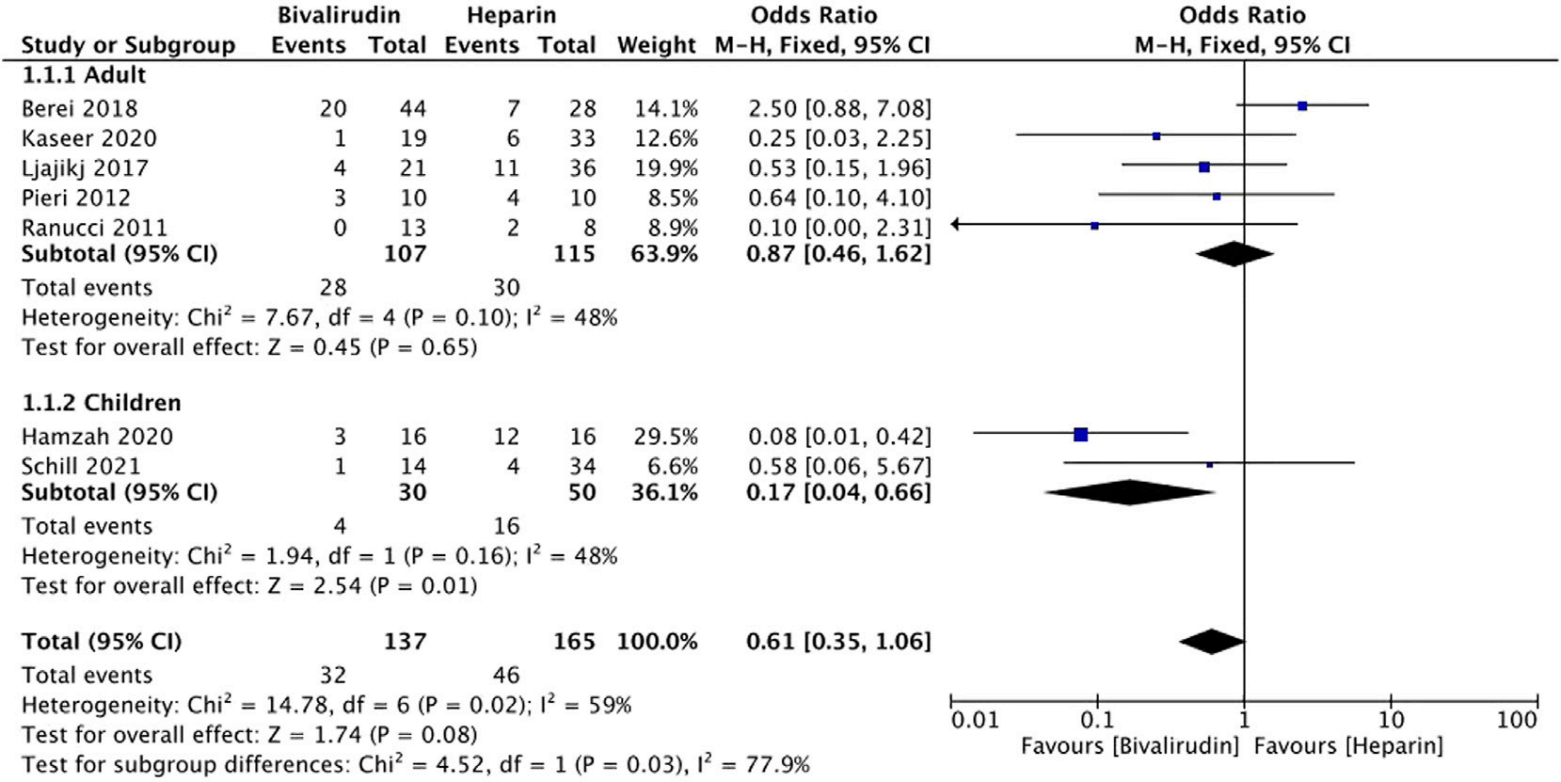
The incidence of major bleeding between the bivalirudin group and the heparin group.

Seven studies (Ranucci et al., 2011; Pieri et al., 2013; Ljajikj et al., 2017; Berei et al., 2018; Hamzah et al., 2020; Kaseer et al., 2020; Schill et al., 2021) were included in the meta-analysis, and moderate heterogeneity was observed (*I* ^*2*^ = 59%, *p* = 0.02), therefore a subgroup analysis was conducted. Five studies (Ranucci et al., 2011; Pieri et al., 2013; Ljajikj et al., 2017; Berei et al., 2018; Kaseer et al., 2020) were included in the adults group, whilst two studies (Hamzah et al., 2020; Schill et al., 2021) were include in the children group (Figure 2). Low heterogeneity was observed in both adults and children group (*I* ^*2*^ = 48% and 48%, *p* = 0.10 and 0.16, respectively), therefore a fixed-effects model was used. The results showed that the difference of pooled incidence of major bleeding was significantly reduced in children (*I* ^*2*^ = 48%, *p* = 0.01, odd ratio (OR) = 0.17, 95% confidence interval (CI): 0.04–0.66) in the bivalirudin group, but not in adults (*I* ^*2*^ = 48%, *p* = 0.65, OR = 0.87, 95% CI: 0.46–1.62). The heterogeneity decreased after subgroup analysis, which indicates that the age maybe one of the sources of heterogeneity.

The sensitivity analysis of the incidence of major bleeding of the included studies showed that all studies’ estimate was within 95% CI of the total effect except for Berei et al. (Berei et al., 2018)’s study, which means the analytical stability may be affected (Supplementary Figure S1). After removing the study the difference of pooled incidence of major bleeding was still not significantly reduced in the adult group (*I* ^*2*^ = 0%, *p* = 0.05, OR = 0.40, 95% CI: 0.16–0.99).

### Thrombosis

Ten studies (Ranucci et al., 2011; Pieri et al., 2013; Ljajikj et al., 2017; Berei et al., 2018; Macielak et al., 2019; Brown et al., 2020; Hamzah et al., 2020; Kaseer et al., 2020; Schill et al., 2021; Seelhammer et al., 2021) reported the incidence of thrombosis. Two studies (Macielak et al., 2019; Brown et al., 2020) were not included due to the different ways of expression (per ECMO day). The incidence rate of thrombosis is 0.207 and 0.089 per ECMO day in the UFH and bivalirudin group in Macielak et al. (Macielak et al., 2019)’s study, 0.043 and 0 per ECMO day in Brown et al. (Brown et al., 2020)’s study, respectively.

**FIGURE 3:**
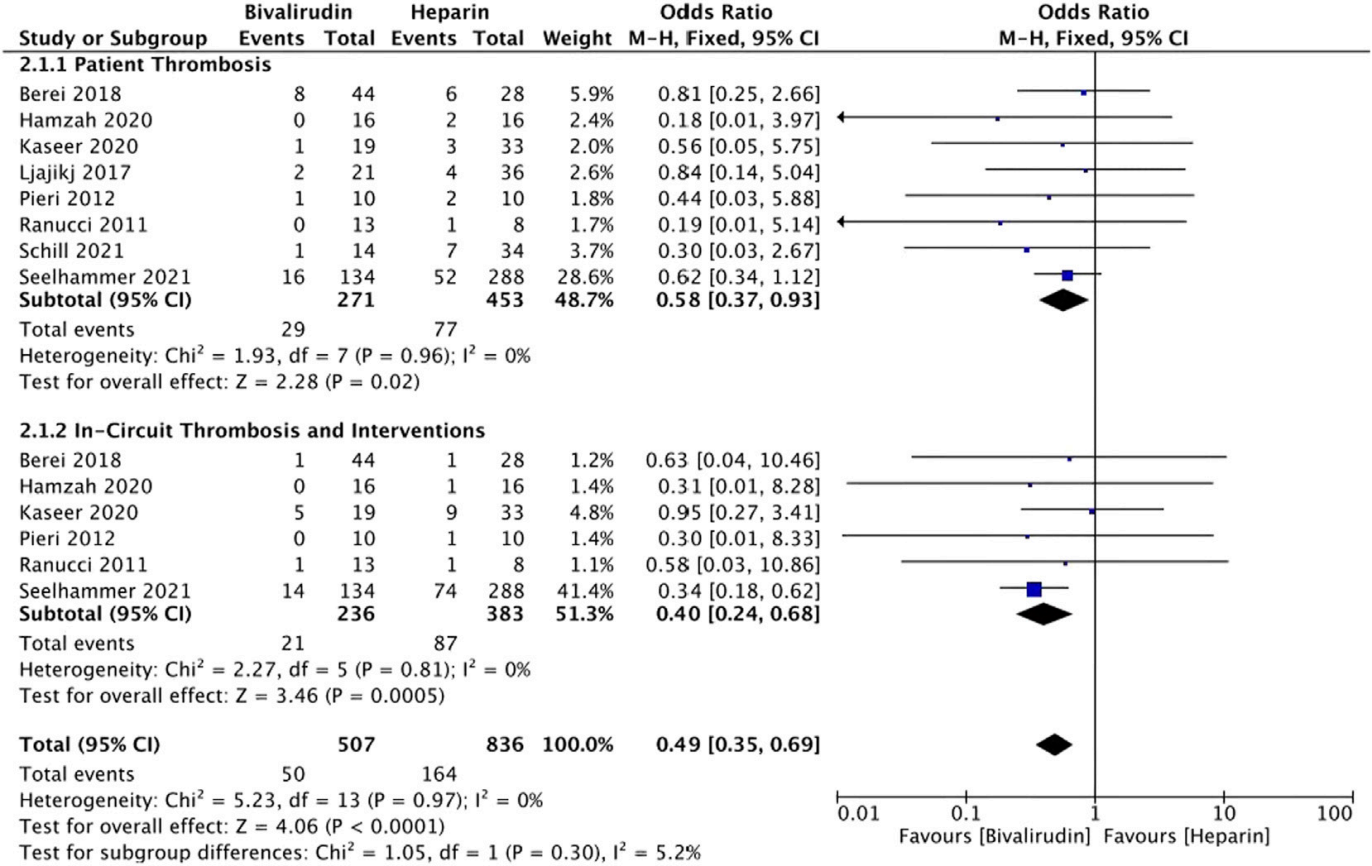
The incidence of thrombosis between the bivalirudin group and the heparin group.

Thrombosis can be divided into patient thrombosis and in-circuit thrombosis/interventions. Eight studies (Ranucci et al., 2011; Pieri et al., 2013; Ljajikj et al., 2017; Berei et al., 2018; Hamzah et al., 2020; Kaseer et al., 2020; Schill et al., 2021; Seelhammer et al., 2021) reported the incidence of patient thrombosis and six studies (Ranucci et al., 2011; Pieri et al., 2013; Berei et al., 2018; Hamzah et al., 2020; Kaseer et al., 2020; Seelhammer et al., 2021) reported the incidence of in-circuit thrombosis/interventions group (Figure 3). Low heterogeneity was observed in both group (*I* ^*2*^ = 0%, *p* = 0.96 and 0.81, respectively), therefore a fixed-effects model was used. The results showed that both the difference of pooled incidence of patient thrombosis (*I* ^*2*^ = 0%, *p* = 0.02, OR = 0.58, 95% CI: 0.37–0.93) and in-circuit thrombosis/interventions (*I* ^*2*^ = 0%, *p* = 0.0005, OR = 0.40, 95% CI: 0.24–0.68) was significantly reduced in the bivalirudin group. The sensitivity analysis showed that all studies’ estimate was within 95% CI of the total effect, which means the analytical stability was not affected (Supplementary Figure S2)

### Mortality

**FIGURE 4:**
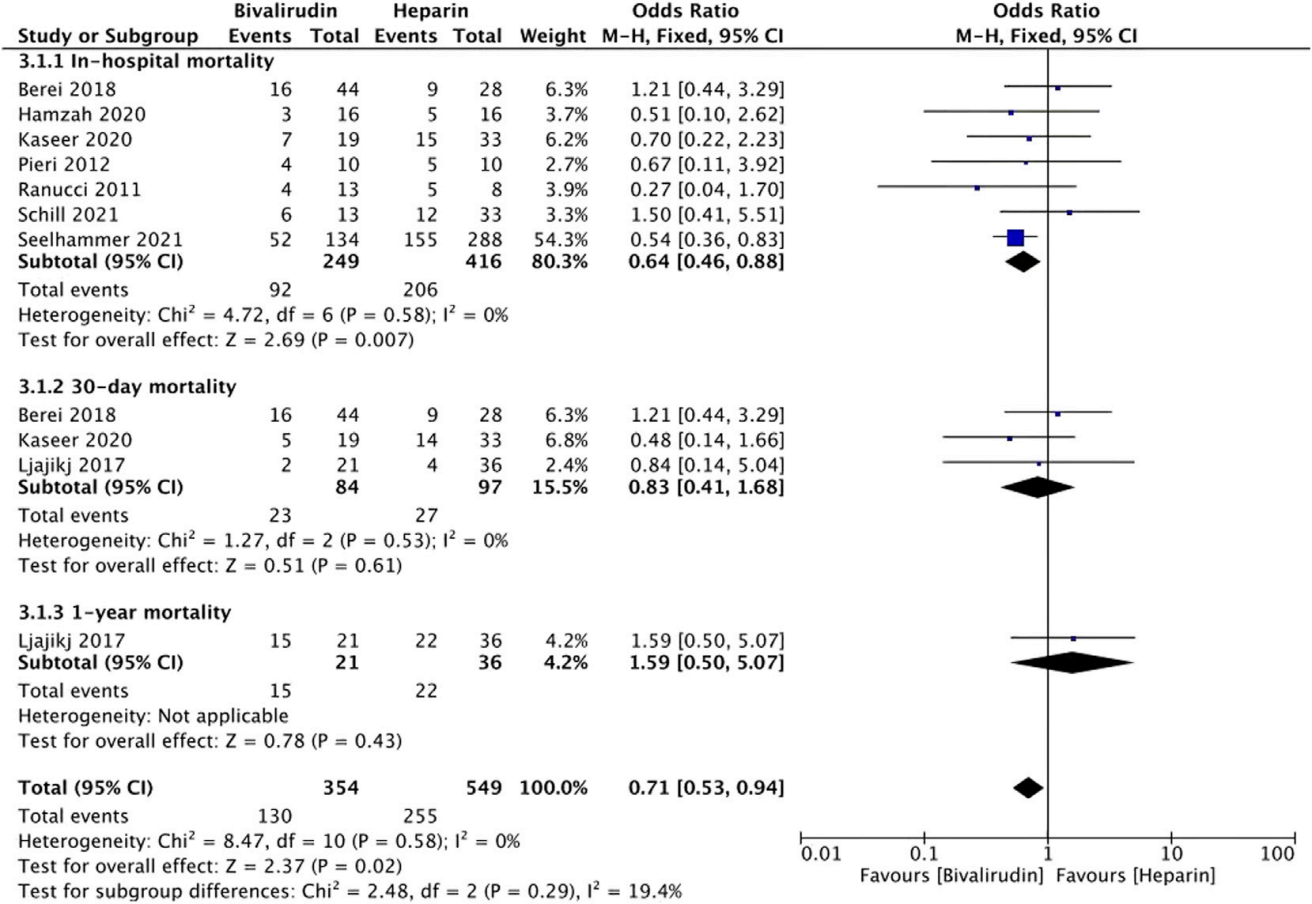
The incidence of mortality between the bivalirudin group and the heparin group.

Seven studies (Ranucci et al., 2011; Pieri et al., 2013; Ljajikj et al., 2017; Berei et al., 2018; Hamzah et al., 2020; Kaseer et al., 2020; Schill et al., 2021; Seelhammer et al., 2021) reported the incidence of mortality. Mortality can be divided into in-hospital mortality, 30-day mortality, and 1-year mortality. Seven studies (Ranucci et al., 2011; Pieri et al., 2013; Berei et al., 2018; Hamzah et al., 2020; Kaseer et al., 2020; Schill et al., 2021; Seelhammer et al., 2021) were included in the in-hospital mortality group, three studies (Ljajikj et al., 2017; Berei et al., 2018; Kaseer et al., 2020) were included in the 30-day mortality group, and only one study (Ljajikj et al., 2017) was included in the 1-year mortality group (Figure 4). Low heterogeneity was observed in both in-hospital mortality and 30-day mortality group (*I* ^*2*^ = 0%, *p* = 0.58 and 0.53, respectively), therefore a fixed-effects model was used. The results showed that the difference of pooled incidence of in-hospital mortality was significantly reduced in the bivalirudin group (*I* ^*2*^ = 0%, *p* = 0.007, OR = 0.64, 95% CI: 0.46–0.88), but the difference of pooled incidence of 30-day mortality was not significant (*I* ^*2*^ = 0%, *p* = 0.61, OR = 0.83, 95% CI: 0.41–1.68). The sensitivity analysis showed that all studies’ estimate was within 95% CI of the total effect, which means the analytical stability was not affected (Supplementary Figure S3)

### ECMO Duration

**FIGURE 5:**
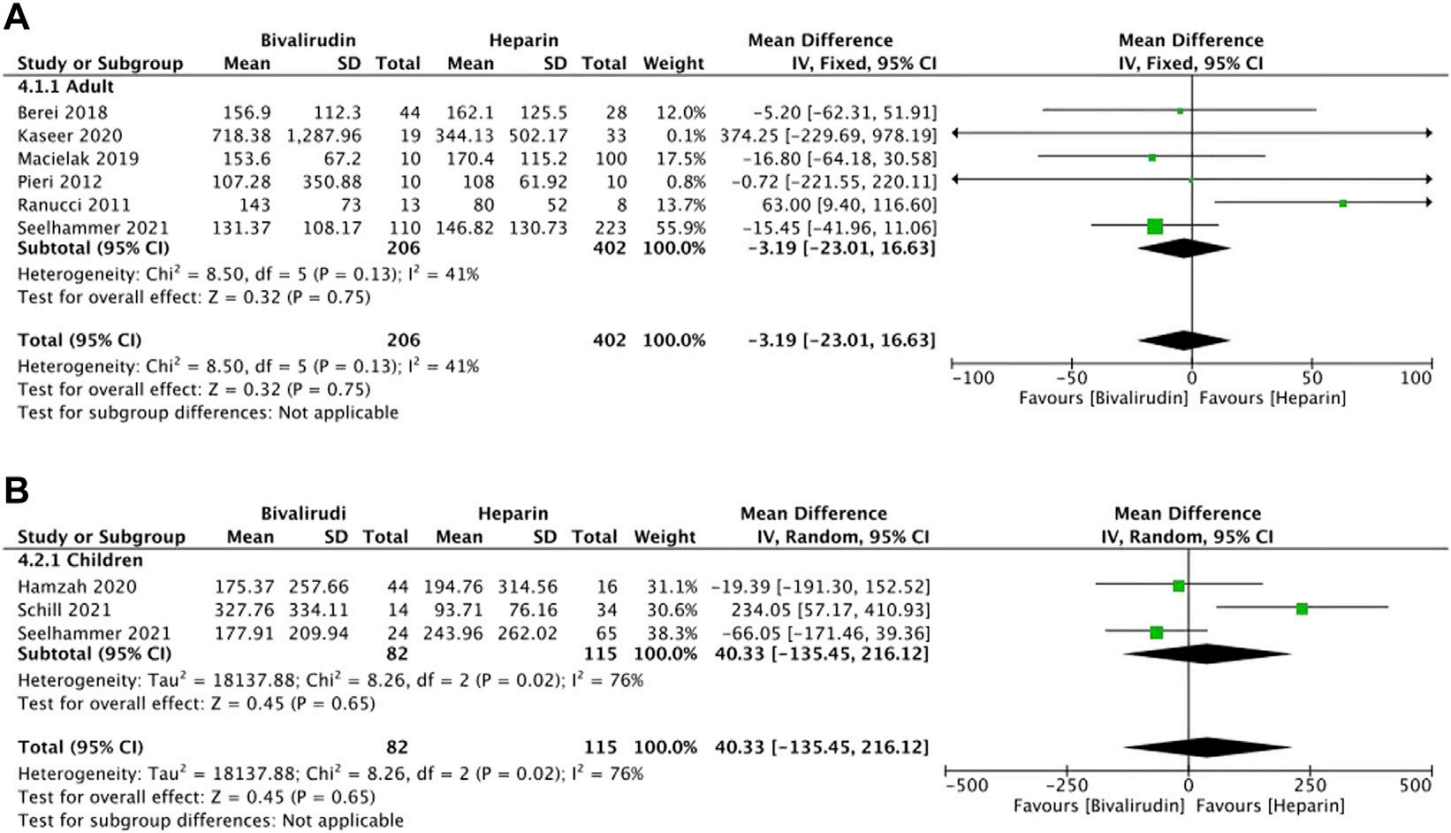
The ECMO duration between the bivalirudin group and the heparin group. **(A)** adult group; **(B)** children group.

Eight studies (Ranucci et al., 2011; Pieri et al., 2013; Berei et al., 2018; Macielak et al., 2019; Hamzah et al., 2020; Kaseer et al., 2020; Schill et al., 2021; Seelhammer et al., 2021) reported the ECMO duration, five of which (Pieri et al., 2013; Hamzah et al., 2020; Kaseer et al., 2020; Schill et al., 2021; Seelhammer et al., 2021) described the ECMO duration between the bivalirudin group and the heparin group *via* IQR (Figure 5). Six studies (Ranucci et al., 2011; Pieri et al., 2013; Berei et al., 2018; Macielak et al., 2019; Kaseer et al., 2020; Seelhammer et al., 2021) were included in the adult group and three studies (Hamzah et al., 2020; Schill et al., 2021; Seelhammer et al., 2021) were included in the children group. Low heterogeneity was observed in the adult group (*I* ^*2*^ = 41%, *p* = 0.13), therefore a fixed-effects model was used. The results showed that the MD of pooled ECMO duration was not significant between the two groups (*I* ^*2*^ = 41%, *p* = 0.75, MD = -3.19, 95% CI: -23.01–16.63).

High heterogeneity was observed in the children group (*I* ^*2*^ = 76%, *p* = 0.02), therefore a random-effects model was used. The results showed that the MD of pooled ECMO duration was not significant between the two groups (*I* ^*2*^ = 76%, *p* = 0.65, MD = 40.33, 95% CI: -135.45–216.12).

After removing the study conducted by Schill et al. (2021), the heterogeneity of this outcome decreased significantly (*I* ^*2*^ = 0%, *p* = 0.68), which indicated the main source of heterogeneity. Therefore, a fixed-effects model was used. The results showed that the MD of pooled ECMO duration was not significant between the two groups (*I* ^*2*^ = 0%, *p* = 0.25, MD = -53.30, 95% CI: -143.16–36.56). However, a directional change occurred after removing Schill et al. (2021), indicating that the results of this meta-analysis maybe not that stable, more studies should be included. For adults’ ECMO duration, the sensitivity analysis showed that all studies’ estimate was within 95% CI of the total effect, which means the analytical stability was not affected (Supplementary Figure 4A). For children’s ECMO duration, the sensitivity analysis showed that two studies’ (Schill et al., 2021; Seelhammer et al., 2021) estimate was not within 95% CI of the total effect, which means the analytical stability maybe affected (Supplementary Figure 4B).

### Publication Bias

Egger’s and Begg’s tests suggested no significant publication bias of the incidence of major bleeding (Egger *p* = 0.093 and Begg *p* = 0.368), patient thrombosis (Egger *p* = 0.116 and Begg *p* = 0.035), circuit thrombosis (Egger *p* = 0.503 and Begg *p* = 0.452), in-hospital mortality (Egger *p* = 0.551 and Begg *p* = 0.764), 30-day mortality (Egger *p* = 0.757 and Begg *p* = 1), ECMO duration in adults (Egger *p* = 0.156 and Begg *p* = 0.133) and children (Egger *p* = 0.282 and Begg *p* = 0.296).

## Discussion

Though no large-scale clinical trials have compared the prognosis of anticoagulation therapy with bivalirudin or UFH, bivalirudin has become the first-line anticoagulant therapy strategy for patients with HR, HIT, or those who need surgery. It is widely used in patients undergoing high-risk percutaneous coronary intervention (PCI) and transcatheter aortic valve implantation (TAVI) (Stone et al., 2006; Kastrati et al., 2008; Stone et al., 2008; Han et al., 2015; Ahmad et al., 2017; Villablanca et al., 2017). To our knowledge, this is the first registered meta-analysis exploring the efficacy and safety of bivalirudin versus UFH in anticoagulation therapy in ECMO. The results showed that bivalirudin can significantly reduce the incidence of major bleeding in children, thrombosis in both patients and pumps, and in-hospital mortality. Also, comparable clinical outcomes were observed in the incidence of major bleeding in adults, 30-day mortality, and ECMO duration.

There are great challenges in treating patients receiving ECMO, and finding the balance between anticoagulation therapy and hemorrhagic complications is essential. Major bleeding is one of the most common complications of ECMO, often affecting the mortality of the patients. We found that bivalirudin can reduce the incidence of major bleeding in children, this is the same in Hamzah et al. (Hamzah et al., 2020)’s study. This phenomenon may due to the reason that children’s livers are immature, and their anticoagulant proteins are defective. What is more, children are more prone to develop HR. Hamzah et al. (Hamzah et al., 2020) observed a shorter time to reach treatment anticoagulation levels and fewer bleeding events in the bivalirudin group than that in the UFH group. As an anticoagulant, UFH can stimulate platelet activation *in vivo*, while bivalirudin can be used as an inhibitor of thrombin-dependent platelet activation and collagen-induced platelet procoagulant activity (Busch et al., 2009; Kimmelstiel et al., 2011). Bivalirudin has better antithrombotic and anticoagulant effects than UFH, with less platelet activation and consumption (Burstein et al., 2019). This may explain children’s lower tendency of major bleeding in the bivalirudin group.

Although low-dose UFH seems to safely reduce the risk of major bleeding and not increase the risk of thrombosis (Carter et al., 2019; Wood et al., 2020), it may not be practical in patients with HIT and HR. As a way to reduce the UFH dose, a heparin-coating circuit can reduce coagulation activation and the inflammatory reaction, protect platelets and coagulation factors, improve biocompatibility, avoid high-dose systemic heparinization, and reduce the dosage of UFH. Nevertheless, studies have reported that the release of UFH from the circuit may also be responsible for HIT, even in small quantities (Pappalardo et al., 2009). In some department protocols, the heparin-coating circuit’s use was continued even after the diagnosis of HIT (Koster et al., 2000). These findings highlight the pitfalls of UFH and the strengths of bivalirudin. The consumption of platelet and thrombin may lead to consumption coagulopathy, which causes intravasular and extravascular thrombosis. Additionally, the complexity of pharmacokinetic parameters may increase due to the increase of volume distribution and random adsorption of drugs on different parts of the pump, which requires continuous dose titration of UFH (Kato et al., 2021). Compared with UFH, bivalirudin has a more predictable pharmacokinetic profile, a greater reduction in thrombin, and no associated incidence of HIT (Netley et al., 2018). HIT can leads to death in some severe cases (Zhong et al., 2020), which greatly affects the in-hospital mortality. This explains the lower in-hospital mortality in the bivalirudin group.

The activated partial thromboplastin time (APTT) value reflects anticoagulation condition: the higher the values, the higher the risk of bleeding. Kaseer et al. found that compared with UFH, the percentage of time that the APTT was within the therapeutic range was higher with bivalirudin (50 vs. 85.7%; *p* = 0.007), which means that bivalirudin more consistently maintained the APTT within the therapeutic range in comparison to UFH. Bivalirudin appears to be a reliable alternative anticoagulation option in patients with pediatric ECMO who have failed UFH (Cuker et al., 2018). The researchers recommended an initial bivalirudin dose of 2.5 mcg/kg/min for all patients, checking the APTT 2 hours after the initial dose and then every 4 hours after that (Netley et al., 2018). However, the optimal monitoring strategy remains to be explored (Ryerson et al., 2020). To monitor bivalirudin therapy, APTT hepzyme (HPTT), intrinsic coagulation pathways with heparinase (HEPTEM), and measurement of the clotting time is recommended (Teruya et al., 2020).

Economic factors should also be taken into consideration as comparable clinical outcomes of the incidence of major bleeding in adults, 30-day mortality, and ECMO duration in both groups. For patients with acute myocardial infarction, treatment with bivalirudin may be a cost-effective option rather than heparin plus glycoprotein IIb/IIIa inhibitor (Schwenkglenks et al., 2011; Schwenkglenks et al., 2012). This cost-effectiveness may translate into the ECMO population as well. In ECMO anticoagulation therapy, although bivalirudin is much more expensive than UFH (reportedly $1170 per vial) (Kaseer et al., 2020), the total cost might be lower due to less frequent monitoring, platelet transfusion, etc (Hamzah et al., 2020; Kaseer et al., 2020). Furthermore, Ranucci et al. also reported that the bivalirudin group lost less blood (*p* = 0.015), and therefore required fewer platelet concentrates (*p* = 0.008), fresh frozen plasma (*p* = 0.02), and purified antithrombin (*p* = 0.048). Thus, the daily cost of ECMO was significantly lower in the patients in the bivalirudin group (Ranucci et al., 2011).

Our study indicated that bivalirudin may provide superior anticoagulation therapy in ECMO compared to that of UFH. For the incidence of major bleeding and thrombosis, patients who received bivalirudin or any other DTI may have done so because of the HIT potential, a hypercoagulable syndrome already predisposing patients to worse outcomes, especially regarding potential thrombotic sequela. For mortality, the underlying cause of patients requiring ECMO is likely the major determinant of outcomes and is already associated with an extremely high risk of adverse events. For the ECMO duration, weaning from ECMO differs between centers, and there is no specific information about standardized weaning protocols (Lüsebrink et al., 2020), more studies are truly requested. From our perspective, to rationally use bivalirudin in ECMO, the baseline APTT value, the presence of renal and/or liver insufficiency, the use of other drugs (e.g., argatroban) (Geli et al., 2021), the possibility of bivalirudin resistance, and the methods of operation should all be taken into consideration.

### Strengths and Limitations

Compared with the former systematic review (Sanfilippo et al., 2017), our study introduced new clinical studies and expanded the sample size, and we conducted the first meta-analysis. However, there are still some limitations in our study. Initially, the studies included were retrospective small-size studies, which means the argumentation intensity may not be strong enough, and only a hypothesis can be generated. We hope that there will be more large-scale RCTs in the future. Secondly, though sensitivity and subgroup analyses were performed, the patients’ variable character may lead to heterogeneity, which may affect the stability of the results. Future research is essential to ensure the homogeneity of the population as much as possible. Finally, the lack of specific results or available research data may restrict our subgroup analysis, such as the use of bivalirudin versus UFH in a different type of ECMOs (VV or VA), and different indications (STEMI, ARDS, heart transplantation, and so forth); these can be further investigated in future studies and enrolled in the sensitivity analysis. Despite these limitations, our meta-analysis provides valuable insight into the use of bivalirudin in the anticoagulation therapy of ECMO.

## Conclusion

Bivalirudin can significantly reduce the incidence of major bleeding in children, patient thrombosis, in-circuit thrombosis/interventions, and in-hospital mortality. Though comparable clinical outcomes were observed in the incidence of major bleeding in adults, 30-day mortality, and ECMO duration, the incidence of the aforementioned complications is seemingly lower in the bivalirudin group. Compared with UFH, bivalirudin is safer, more practical, and dependable, which can be a safe and feasible alternative anticoagulant option to UFH as anticoagulation therapy in ECMO, especially for patients at risk for HR and HIT.

## Data Availability

The original contributions presented in the study are included in the article/Supplementary Material, further inquiries can be directed to the corresponding authors.
